# Soil Salinity, a Serious Environmental Issue and Plant Responses: A Metabolomics Perspective

**DOI:** 10.3390/metabo11110724

**Published:** 2021-10-22

**Authors:** Kekeletso H. Chele, Morena M. Tinte, Lizelle A. Piater, Ian A. Dubery, Fidele Tugizimana

**Affiliations:** 1 Department of Biochemistry, University of Johannesburg, Auckland Park, Johannesburg 2006, South Africa; ckekeletso@gmail.com (K.H.C.); morenatinte@gmail.com (M.M.T.); lpiater@uj.ac.za (L.A.P.); idubery@uj.ac.za (I.A.D.); 2 International Research and Development Division, Omnia Group, Ltd., Johannesburg 2021, South Africa

**Keywords:** environmental factors, salinization, abiotic stresses, metabolomics, salinity

## Abstract

The effects of global warming have increasingly led to devastating environmental stresses, such as heat, salinity, and drought. Soil salinization is a serious environmental issue and results in detrimental abiotic stress, affecting 7% of land area and 33% of irrigated lands worldwide. The proportion of arable land facing salinity is expected to rise due to increasing climate change fuelled by anthropogenic activities, exacerbating the threat to global food security for the exponentially growing populace. As sessile organisms, plants have evolutionarily developed mechanisms that allow ad hoc responses to salinity stress. The orchestrated mechanisms include signalling cascades involving phytohormones, kinases, reactive oxygen species (ROS), and calcium regulatory networks. As a pillar in a systems biology approach, metabolomics allows for comprehensive interrogation of the biochemistry and a deconvolution of molecular mechanisms involved in plant responses to salinity. Thus, this review highlights soil salinization as a serious environmental issue and points to the negative impacts of salinity on plants. Furthermore, the review summarises mechanisms regulating salinity tolerance on molecular, cellular, and biochemical levels with a focus on metabolomics perspectives. This critical synthesis of current literature is an opportunity to revisit the current models regarding plant responses to salinity, with an invitation to further fundamental research for novel and actionable insights.

## 1. Introduction: Problem of Soil Salinization and Impact of Salinity on Plants

The food and agriculture organisation (FAO) has reported climate change as one of the greatest challenges facing farmers and the entire agricultural sector in the 21st century [1]. In addition to exponential population growth, the negative impacts of unpredictable climate changes due to global warming pose a great threat to agricultural sustainability. Furthermore, the effects of global warming have increasingly led to devastating environmental stresses, such as extreme temperatures, salinity, drought, and floods, which exacerbate declines in crop productivity, quantity, and quality [2,3]. Salinity is one of the top detrimental abiotic stresses, affecting 7% of land area and 33% of irrigated lands worldwide. According to the latest studies, the frontiers of salt-affected land include Australia, North and Central Asia, and South America, with a distribution of 357, 211, and 129 million hectares of area under salinity, respectively [4]. In addition, the agricultural sector has estimated an annual loss of 27.3 billion US dollars due to agricultural damages caused by saline soils [5,6]. Thus, in addition to global warming effects and other environmental factors, the presence of excessive salts in soil is a foremost challenge to global food security for the exponentially growing world population.

Salinity stress has been defined as the accumulation of salts in the rhizosphere, predominantly sodium (Na^+^) and chloride (Cl^−^) ions [7,8]. Generally, soil is considered saline when the electrical conductivity (EC) of the saturation extract in the root zone exceeds 40 mM at 25 °C, with 15% of unbound Na^+^ ions [9]. Currently, studies have reported an estimate of 33% of irrigated agricultural lands and 20% of the cultivated lands that are highly saline, with an expected increase of 10% annually [10,11]. These soil salts have been aggravated by overuse of chemical fertilizers, irrigation malpractices, industrial pollution, gradual withdrawal from the ocean, as well as mineral weathering. Some fertilizers contain high levels of salts and reduced water holding capacity, leading to a greater salt concentration in the soil upon evaporation [12,13].

While soil salinisation can be a result of anthropogenic acts, such as agricultural malpractices, it is being accentuated by rising sea levels and water intrusion from the sea to arable lands [9,14]. In the last century, sea levels have risen faster than they have in the past 3000 years and are expected to rise a further 15–25 cm by 2050 due to global warming. Consequently, soil salinity will continue to be an enormous environmental problem in the foreseeable future, with an estimated 50% of salinized arable land by 2050 [6]. According to the Intergovernmental Science-Policy Platform on Biodiversity and Ecosystem Services (IPBES) and the U.S. Agency for International Development (USAID), the estimated measure of salt-affected regions has risen from 45 million hectares to 62 million hectares since the 1990s, making salinity the major factor in the reduction of plant growth and productivity globally [6,15].

Several plant sciences studies have reported that soil in dry areas is naturally very salty, hence poor irrigation practices and drainage leads to an increase of the water table, bringing the salts towards the surface. When the water evaporates, the salt ion residue is left around the roots, inhibiting the uptake of water and other nutrients, hence stunting plant development at all stages, including germination, vegetative growth, and the reproductive stage [16,17]. The salinity-impacted soils produce agricultural crops exhibiting a spectrum of complex responses and interactions of physiological, morphological, and biochemical processes, ultimately leading to very low crop production and quality [18]. In addition, soil salinization not only affects crop production, but the physiochemical properties and the ecological balance of the affected areas [9]. Although most crop plants are susceptible to salinity, halophytes are a special group of plants that survive in highly salinized areas. The study of halophytes can thus advance our understanding of the crucial adaptations required for high salinity tolerance, including the secretion of salt, osmotic adjustments, and regulation of ion homeostasis.

Thus, the urgency of securing food supply for the growing populace under unfavourable agricultural conditions, such as salinization, has inspired the prioritisation of plant-environment interactions research. Knowledge and comprehensive understanding of the complex mechanisms governing salinity tolerance in plants is imperative for improved crop productivity, and hence food security. Understanding the physiological and biochemical responses to salinity stress could provide significant strategies to improve crop tolerance to salinity stress. Thus, this review aims to summarize the various mechanisms regulating salinity stress tolerance on molecular, cellular, and biochemical levels with a focus on *omics* perspectives, particularly metabolomics. To comprehensively articulate the devastating effects of salinization on crop production, this review presents the problem and impact of salinization on crop plants, the subsequent plant responses to salinity stress, and the use of metabolomics as a technique to unravel the mechanisms underlying plant responses to salinity. As the global qualitative and quantitative analyses of metabolites, metabolomics provides comprehensive insights of cellular activities, thus revealing functional signatures of the biochemical landscape and cellular physiology of the system under consideration.

## 2. Plant Responses to Salinity Stress: Cellular and Molecular Events

**Figure 1:**
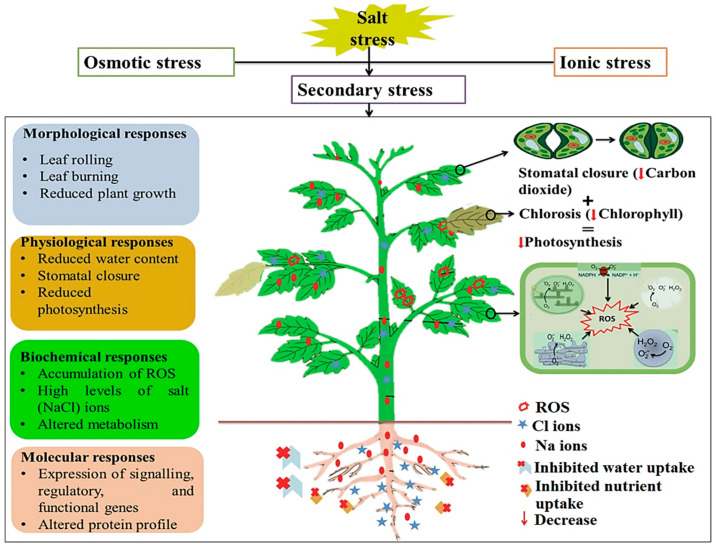
Summarised morphological, physiological, biochemical, and molecular responses to salinity stress. Accumulation of ions, mainly sodium (Na^+^) and chloride (Cl^−^) ions, in the soil inhibit water and nutrient absorption, leading to impaired cellular water status (osmotic stress) and an extreme case of ion toxicity. In addition to an overproduction of macromolecule-damaging reactive oxygen species (ROS), the resultant secondary effects of salinity stress entail stomatal closure, reduced water content, and chlorosis, hence a reduced rate of photosynthetic activity.

Salinity stress manifests in two main phases: the osmotic phase and the late ionic phase (Figure 1). Both phases can result in the occurrence of secondary stresses, most commonly oxidative stress due to excess production of reactive oxygen species (ROS) [18,19,20]. Excessive salts in the soil reduce water uptake by plants due to reduced osmotic potential at the root surface. This characterizes the early osmotic stress stage, which leads to stomatal closure and reduced shoot growth (Figure 1). In the late ionic phase, the accumulation of several ions including K^+^, Zn^2+^, Mn^2+^, and Mo^2+^ ions in cells (ionic stress) results in toxic effects, including disruption of membrane structures and cellular organelles, impaired uptake of beneficial nutrients, change in photosynthetic and transpiration rates, leaf senescence, and hindered enzymatic activity (Figure 1) [16,21]. In addition to the salinity stress-induced changes in the ionome of a plant, salt stress, mostly resulting in ion toxicity, can lead to an impediment in photosynthesis (mainly in salt-susceptible plants) due to a reduction in the chlorophyll content—a chlorosis condition. The latter is symptomatically characterized by leaves that are pale, yellow, or yellow-white. Furthermore, the affected plant is unable to synthesize carbohydrates through photosynthesis, which negatively impacts on the health, growth, and development of the plant [22]. Ultimately, both salinity stress phases affect molecular, morphological, physiological, and biochemical processes in plants, resulting in growth suppression and, failing stress alleviation, cell death.

For their survival against the aforementioned unfavourable conditions, plants have evolved a well-coordinated defence system to sense and respond to stress factors [23,24]. At cellular and molecular levels, plant defence is characterised by highly regulated and multi-layered complex mechanisms that span a series of events governing stress tolerance. These involve stress signal perception and transduction, and a reprogramming of the genetic, transcriptomic, and metabolic machineries, which are translated into biochemical and physiological stress-responsive phenomenology [25]. Several studies have delved into the impacts of salinity stress, from the regulation of gene expression to the coordination of signalling, regulatory, and metabolic pathways [16,26,27]. As with several other abiotic stresses, salinity stress is perceived by cell surface-localised damage-associated molecular patterns (DAMPs), which lead to phosphorylation reactions responsible for activation of the nicotinamide adenine dinucleotide phosphate (NADP) oxidase, an enzyme that catalyses production of ROS [28]. Simultaneously, the accumulation of salt ions also stimulates excessive release of abscisic acid (ABA), which has roles in the activation of downstream stress signalling and response cascades [29].

### 2.1. Signalling Pathways in Salinity Stress Conditions

Salinity stress-signal transduction mechanisms can be summarised in a few most notable pathways: calcineurin B-like protein (CLB)—CBL-interacting protein kinase (CIPK), salt overly sensitive (SOS), calcium-dependent protein kinase (CDPK), mitogen-activated protein kinase (MAPK), and hormone signalling pathways [30,31]. In cooperation with the second messengers, ROS and Ca^2+^, these pathways lead to the activation of defence responses [32].

#### 2.1.1. Second Messenger Signalling: ROS and Ca^2+^ Waves

Amongst the most intensively studied signalling second messengers are ROS and Ca^2+^ ions, which serve as signatures for both signalling and cell-to-cell communication [33,34,35]. ROS comprise reactive forms of molecular oxygen, including hydrogen peroxide (H_2_O_2_), superoxide radical (O_2_^−^), singlet oxygen (^1^O_2_), and a hydroxyl radical (OH^−.^) [36]. Plants have adapted a built-in antioxidant machinery that comprises three main enzymes: superoxide dismutase (SOD), ascorbate peroxidase (APX), and catalase (CAT) to prevent the damaging effects of ROS accumulation [37,38,39]. In addition, non-enzymatic antioxidant molecules like ascorbate, glutathione, and carotenoids assist in reduction of the oxidative damage resulting from the oxidative burst [40,41,42]. ROS production and scavenging is compartmentalised; hence, different abiotic stresses can induce ROS production in numerous cell compartments (chloroplasts, mitochondria, peroxisomes) either directly or indirectly, via the action of other signalling pathways [43,44].

**Figure 2:**
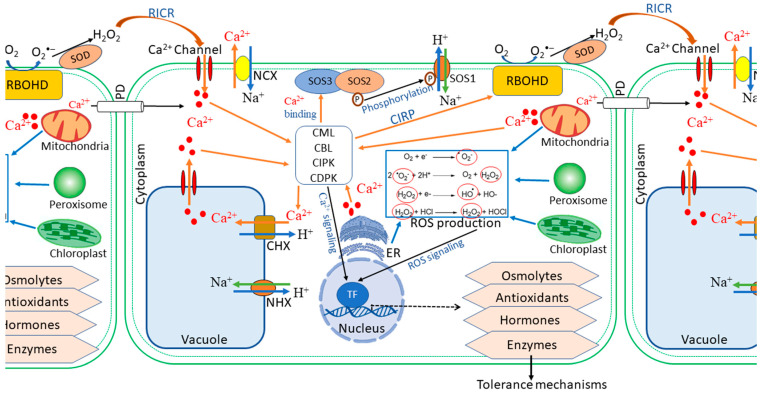
The interplay of reactive oxygen species (ROS) and calcium ions (Ca^2+^) in plant signalling. ROS-induced Ca^2+^ release (RICR): accumulation of ROS induces release of Ca^2+^ through Ca^2+^ channels; in turn, the Ca^2+^ ions enhance the production of ROS through activation of the RBOHD membrane protein in the process called Ca^2+^-induced ROS production (CIRP). The superoxide produced by RBOHD is dismutated to hydrogen peroxide by the scavenging enzyme, superoxide dismutase (SOD). Activation of RBOHD proteins in initiating stress-stimulated cells results in overproduction of ROS, which are transported to adjacent cells, which, in addition to the Ca^2+^ entering through the plasmodesmata (PD), trigger calcium release from reserves. The accumulated Ca^2+^ activates the RBOHD to enhance ROS production. In addition, Ca^2+^ activates SOS3 protein, which, in turn, interacts with SOS2 and triggers phosphorylation of SOS1 to increase Na^+^ efflux. Ca^2+^ and Na^+^ signalling ultimately activate transcription factors for the production of stress-responsive molecules with roles in salinity tolerance mechanisms. Abbreviations: CBL, calcineurin B-like protein; CML, calmodulin-like protein; CDPK, calmodulin-dependent protein kinase; CIPK, calcineurin B-like interacting protein kinase; CHX, Ca^2+/^H^+^ exchanger; CICR, calcium-induced calcium release; ER, endoplasmic reticulum; NCX, Na^+^/Ca^2+^ exchanger NHX, Na^+^/H^+^ exchanger; PD, plasmodesmata; RBOHD, respiratory burst oxidase homolog protein, RICR, ROS-induced Ca^2+^ release; ROS, reactive oxygen species; SOS, salt overly sensitive; TF, transcription factor.

Despite the damaging effects, ROS are essential signalling molecules that instigate local defence responses as well as evoke adaptive responses, such as systemic acquired resistance (SAR) and systemic acquired acclimation (SAA) [43,45,46]. Stimulation by excessive salts in the soil initiates an enhanced production of ROS, a process dependent on the activity of the respiratory burst oxidase homolog protein, RBOHD (a superoxide-generating NADPH oxidase) [31,35]. A burst of ROS in affected local tissue triggers a systemic autopropagating wave thereof (Figure 2). The autopropagating nature of the signal suggests that each cell along the path activates its own RBOHD enzymes, which generate ROS capable of triggering all adjacent cells to undergo the same process, thereby relaying the threat signal throughout the entire plant [31,34].

Similarly to ROS, cytosolic calcium serves as a central hub in the signalling network, relaying signals throughout the plant in response to different environmental stimuli [47,48] (Figure 2). Regarding abiotic or hormonal stimuli, a transient and defined pattern of cytosolic calcium level elevation characterises early stage stress response to salinity stress, thereby triggering downstream responses, which involve phosphorylation cascades and regulation of gene expression [48,49]. For signalling in systemic defence and acclimation, the wave of increased cytosolic Ca^2+^ levels, like that of ROS, propagates systemically to the aerial parts of the plant [35,43]. The downstream events of calcium signalling are largely mediated by the calcium-binding proteins, which interact with calcium ions, and eventually lead to altered gene expression and regulation of plant defence responses [50,51]. Calcium-mediated signalling in stress defence comprises two prominent pathways: the CBL-CIPK and CDPK pathways [52,53]. Ultimately, both signalling pathways decode and transmit the Ca^2+^ signals to activate stress-responsive genes, thus leading to an effective response to the stressor stimuli [54].

Previous studies have shown that at least two different functional processes could link ROS and calcium-medicated signalling in plants, namely, Ca^2+^-induced ROS production (CIRP) and ROS-induced Ca^2+^ release (RICR) (Figure 2) [33,34,55]. In CIRP, Ca^2+^ has been shown to activate RBOHD proteins. In contrast, RICR could be mediated directly by ROS-induced activation or suppression of Ca^2+^ channels or pumps [31,35]. At the cellular level, under stressful conditions, the accumulation of Ca^2+^ triggers RBOHD protein activity, hence ROS production, via CBL/CIPK or other Ca^2+^-RBOHD (Figure 2) [35,46,56]. Activation of the RBOHD proteins in the initial cells leads to an accumulation of ROS, which in turn travels to the neighbouring cells. The subsequent ROS wave either activates RICR, or the RBOHD proteins of the adjacent cells could be triggered by the Ca^2+^ from the initiating cells transported through the plasmodesmata (Figure 2) [43,47,57]. In addition to the abovementioned mechanisms, the highly-conserved MAPK cascades are activated to commence signal transduction pathways that lead to diverse cellular process in response to stress stimuli [25,58,59]. This activation finally leads to phosphorylation of downstream substrates including transcription factors, which are responsible for genetic reprogramming orchestration towards plant defence [25,60].

#### 2.1.2. The SOS Signalling Pathway

One of the key responses against salinity stress is the maintenance of cellular ion homeostasis through restriction of the accumulation of Na^+^ in the cytoplasm. A well-defined signal transduction pathway in response to ion toxicity is the salt overly sensitive (SOS) pathway, one of the CBL-CIPK signalling pathways contributing to the plant’s salinity tolerance (Figure 2) [54,61]. The SOS pathway is the primary mechanism used by plant roots to regulate Na^+^ exclusion, thus protecting the plant by delaying the effects of cytoplasmic ion toxicity [62,63]. The SOS pathway consists of three major proteins: SOS1 (Na^+^/H^+^ antiporter), SOS2 (serine/threonine kinase), and SOS3 (Ca^2+^-binding protein). Upon perception of salinity, the sharp increase in Ca^2+^ levels, which binds the SOS3 protein, facilitates the interaction between SOS3 and SOS2, hence resulting in activation of the latter. In turn, the SOS3/SOS2 kinase complex is loaded onto the plasma membrane, where it phosphorylates SOS1, stimulating an increase in Na^+^ efflux, thereby reducing cellular Na^+^ toxicity [63,64].

The regulatory mechanisms of Na^+^ efflux through the SOS pathway are far more sophisticated than our current understanding, and it appears that the linear SOS3-SOS2-SOS1 signalling pathway is not as simple as it seems [61,65]. Although not well understood, previous studies have reported that the Na^+^-induced SOS pathway is mediated by ROS. The connection between ROS signalling and SOS signalling was further demonstrated via the interaction of serine/threonine kinase (SOS2) with nucleoside diphosphate kinase 2 (NDPK2) and catalases for the direct activation of SOS2, in the absence of the SOS3 protein [37,59]. In addition, crosstalk between the SOS pathway and some phytohormones involved in salinity responses have been suggested. Brassinosteroids (BRs) have been reported to induce cytosol accumulation of Ca^2+^, which in turn activates the SOS pathway, while microarray analyses under salinity have revealed dramatically different expression profiles of auxins (AUXs) and ethylene (ET) signalling affected by the SOS2 and SOS3 mutations [41,63]. Moreover, it has been reported that under normal conditions, SOS2 is inhibited by several protein factors, among which is the phosphatase abscisic acid (ABA) insensitive 2 (ABI2), suggesting the involvement of abscisic acid in the regulation of the SOS pathway [62].

#### 2.1.3. Hormonal Crosstalk in Salinity Responses

**Figure 3:**
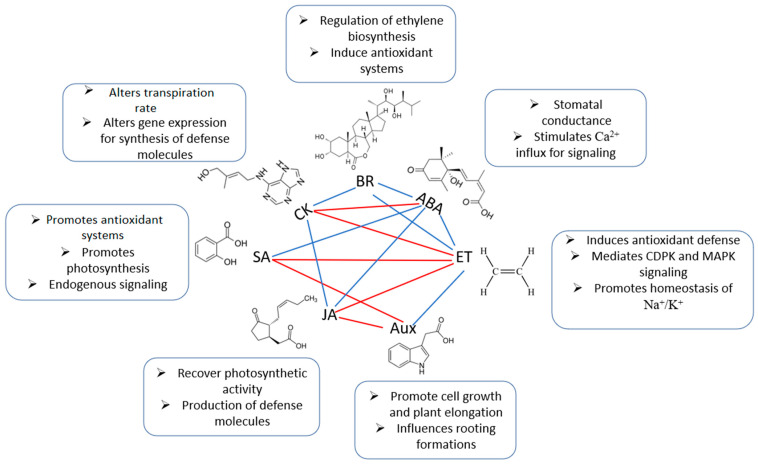
Crosstalk of hormone signalling and their respective roles in response to salinity stress. Phytohormones respond in a complex, coordinated, and interactive manner to alleviate salinity effects and build tolerance. Based on different plant species and the extent of the stress, phytohormones respond with either synergistic (blue lines) or an antagonist (red lines) relationships in their signalling. ABA assumes a predominant position in the complex networking since it interacts with most other plant hormones in stress-responsive signalling pathways. Abbreviations: ABA, abscisic acid; Aux, auxins; BR, brassinosteroids; CK, cytokinins; ET, ethylene; JA, jasmonic acid; SA, salicylic acid.

Plants need to perceive and react to stress stimuli in a highly coordinated and interactive manner to survive the adverse conditions posed by environmental stresses, including salinity. This adaptation is mediated by the ability of various plant hormones and other signalling molecules to interact collectively to finetune defences against environmental challenges [14,63]. The cardinal phytohormones ABA, salicylic acid (SA), jasmonic acid (JA), and ET interact closely with each other and with other stress-response hormones including AUXs, cytokinins (CKs), and BRs, [38,64,65]. The convergence of different signalling pathways is considered crosstalk, which describes a communication network between signalling molecules for a more united and efficient defence signalling (Figure 3) [63,65]. The alterations in the levels of hormones (ABA, CK, ET, JA, SA, AUX, BRs) and second messengers (ROS, Ca^2+^, nitrogen oxide/NO) and subsequent signal transduction cascades have been revealed as the primary salinity responses [65,66,67].

ABA has been found to be the key player in the regulation of signalling pathways, and is the primary hormone involved in the response to many abiotic stresses, including drought, salinity, high heat, metal toxicity, and cold (Figure 3) [68,69]. It has been well-characterized that salinity and the subsequent Ca^2+^-dependent phosphorylation as well as the downstream signalling pathways rapidly activate ABA genes. In turn, elevated ABA levels also upregulate other stress signalling cascades, including the MAPK pathways, which result in downstream expression of genes associated with ET, CKs, BRs, and AUXs. These synergistic interactions lead to subsequent mechanisms responsible for stimulating an influx of extracellular Ca^2+^ for RICR, and/or promote the generation of ROS for CIRP, thus creating ROS and Ca^2+^ waves for signal autopropagation [53,70,71]. Concurrently, ABA also interacts synergistically with JA (Figure 3) and signalling molecules, such as NO, to induce stomatal closure [72,73]. Similar to ABA, the accumulation of JA in salt-stressed plants further activates the release of extracellular Ca^2+^, which stimulates CDPK production, and ultimately results in the activation of stress-related signal cascades [50,74].

Under saline conditions, a significant decline in CK biosynthesis in the roots, hence the consequent supply to the shoots, suggests a role of these phytohormones in plant responses to salinity stimuli [65]. A synergistic relation between CKs and BRs has been reported, where the two phytohormones function coherently to reduce stomatal conductance and facilitate the global gene expression network, thereby eliciting appropriate responses to ameliorate stress responses [75]. Consistently, studies have also provided evidence of the existence of crosstalk between CKs and ABA (Figure 3). While ABA functions to avoid plant stress by promoting stomatal closure to minimize water loss, CKs facilitate responses by delaying both stomatal closure and leaf senescence [18,76]. In addition to CKs, BR-enhanced tolerance has also been attributed to crosstalk with other hormones including ABA and ET. Although the mechanisms are poorly understood, exogenous application of BRs has been reported to result in subsequent elevation of both ABA and ET, leading to an increase in salinity tolerance [77,78].

ET-response factors have been proven to alleviate salinity effects by mediating ROS generation and scavenging events, ultimately causing a decrease in ROS accumulation [67,77]. ET also functions synergistically with ABA by activating transcription factors involved in ABA production, and with AUXs through regulation of root development and architecture, which is a key aspect in salinity tolerance [79]. Stress-induced regulation of AUXs leads to an expansion of root cells along the radial axis in the epidermis and cortex, hence an increase in the length and density of both roots and the root hairs [80,81]. This restructuring of roots therefore leads to enhanced water and nutrient uptake even under high salinity. On the contrary, an antagonist interaction between SA and AUXs has been evidenced by repression of auxin responses as a result of elevated SA signalling [80,82]. Further evidence was provided by the suppression of auxin-responsive genes following induction of exogenous SA [83]. Melatonin, another candidate phytohormone, has been reported in several studies to mediate salinity via direct pathways, such as clearance of ROS, but also indirectly by enhancing antioxidant activity, photosynthetic efficiency, as well as through regulation of stress-related transcription factors [84]. It should be noted that most of the knowledge on hormonal crosstalk in response to salinity is from studies based on the (traditional) model plant, *Arabidopsis*; as such, there might be nuances in the regulatory mechanisms of the hormone-mediated responses in non-model crop plant systems, such as maize, sorghum, and rice [8,85].

As articulated in the above paragraphs, the plant responses to saline conditions are multi-layered and complex events, at both the molecular and cellular levels. However, there are still knowledge gaps in regard to the phenomenology of these plant defences under salinity conditions [81,86]. Current models provide some insights into the activation of signalling events and downstream reconfiguration of hormonal networks, activation of defence-related genes, and rewiring of various metabolic hubs [86,87]. The regulation and coordination of these molecular and cellular events are still enigmatic. Furthermore, the underlying metabolic and biochemical frameworks that define physiological and phenotypic coherence in salinity responses are not fully understood [81,87]. Thus, fundamental interrogation of plant metabolism under salinity is still ongoing, with efforts to provide actionable insights for designing a roadmap for strategies and the next generation of crops for high productivity and resilience to climate change.

## 3. Metabolomics for Elucidation of Plant Responses to Salinity

**Figure 4:**
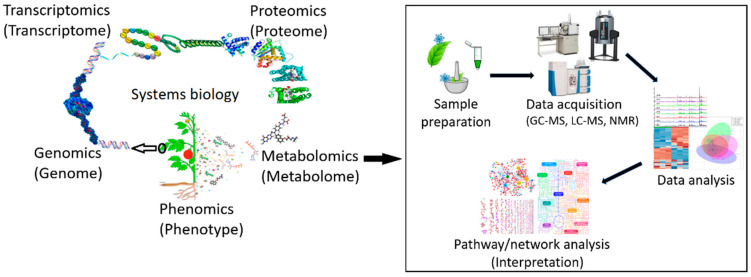
The core *omic* advances in systems biology. As an interdisciplinary field, systems biology provides a holistic view of a biological system via correlation between biomolecules at different organizational levels (genome, transcriptome, proteome, and metabolome) with the emergence of the biological phenomena (phenotype).

Recent advances in the practice of integrating the *omics* techniques have provided new insights and opened innovative perspectives for molecular understanding of the stress stimuli, signal transduction, and transcription regulation in response to abiotic stresses [23,84,88]. Systems biology is a consolidative discipline, which connects the molecular components within and among a single and/or different biological scales (e.g., cells, tissues, and organ systems) to physiological functions and phenotypes through quantitative and computational methodologies [89,90]. Thus, systems biology can be applied in holistically studying the interactions between the genes, regulatory elements (i.e., transcription factors), proteins, and metabolites that are involved in phenotypic/physiological stress adaptations and tolerance in a biological system (Figure 4) [88].

At the epigenetic level, response to salinity stress is classified into three histone modifications: acetylation, methylation, and phosphorylation. Under stringent saline conditions, histone acetylation is controlled by the antagonist action between the histone acetyl transferases (HATs) and histone deacetylases (HDACs), which acts as a positive signal for activation of transcription factors involved in stress responses [91]. Furthermore, the methylation aspect of epigenetic regulation has been reported in pathways responsible for ROS scavenging, including the flavonoid and several other antioxidant biosynthetic pathways. In addition, phosphorylation mechanisms have also been reported to mediate salinity stress response via utilisation of the energy stored in the phosphodiester bond of ATP to remodel the chromatin architecture and induce expression of stress-responsive genes [91,92].

As the assemblage of the downstream products of gene and protein expression, the metabolome represents integrative information across multiple functional levels by mapping genetic reprogramming to different phenotypes, making it a frontier in the systems biology era [93]. The metabolome is understood as the complement of all small-molecular-weight (≤1500 Da) molecules (metabolites) present in a biological matrix. Being a chemical space and language of metabolism, the metabolome carries imprints of environmental and genetic factors [94,95]. Thus, it is expectedly more sensitive to perturbations in both metabolic fluxes and enzyme activity than either the transcriptome or proteome. Hence, one of the most biological descriptions of metabolism is the metabolic profiles and fluxes it generates, which represent the integrated output of the molecular machinery and biochemical characteristics of a biological system [37,69,96,97,98]. Regulation of metabolism encompasses several levels, including transcriptional and post-transcriptional mechanisms, indicating the multi-layered complexity of the processes with end results presented as metabolites [98].

The field of metabolomics is a multidisciplinary *omics* science that has matured and disruptively pushed the traditional boundaries of scientific endeavour by contributing substantially to the data-driven understanding of metabolism under different physiological conditions. As such, metabolomics provides a snapshot of the cellular physiological state and contributes to the understanding of the complex molecular interactions in a biological system [21,91,92]. In recent years, there have been exciting applications of metabolomics in a plethora of plant-environment sciences, including plant-pathogen interactions [99,100], fruit development [101,102], linking genotype and biochemical phenotype [103,104], plant priming [105,106,107], and plant-environment interactions [108,109,110]. In the field of plant responses to stress conditions, metabolomics represents the downstream results of mechanisms from the signal perception to stress tolerance [111,112,113].

To gain insight into the plant responses to salinity, metabolomics is considered a powerful tool that allows the investigation of the complex metabolite changes associated with the salinity response in detail [87]. Such metabolomics studies decipher the mechanisms of osmoadaptation by metabolomic rearrangement, thereby more accurately (compared to other *omics*) reflecting the comprehensive outcomes of gene expressions and regulatory processes involved in plant responses to salinity [114,115]. Such an understanding of the alterations of the plant metabolome under salinity could provide clues to improve salinity tolerance in affected plants, hence optimizing crop productivity and quality in stressed plants [116,117].

### Plant Responses to Salinity: Metabolic Reprogramming

The tolerance ability of plants under salinity is typically based on their capacity to regulate the levels of several stress-responsive mechanisms, including the aforementioned signalling cascades involving second messengers, phytohormones, and the alterations of the metabolic profiles [9,25]. Thus, adaptation and acclimatisation of plants to saline conditions require the establishment of a new state of cellular homeostasis, which implies a reconfiguration of metabolic networks to maintain essential metabolism, cellular plasticity, and rebalancing various physiological processes [118]. As with several other abiotic stresses, salinity can lead to the hyperaccumulation of a wide range of metabolites in plants, and this alteration of the plant metabolome essentially leads to salinity tolerance in affected plants [119,120]. However, these responses to salinity conditions are highly complex and depend on the stress intensity and extent, the developmental stage and growth conditions, as well as the genetic and metabolic predispositions of the plant under consideration [26,87].

Plant metabolism is defined by the primary (central) metabolism, which entails pathways absolutely critical for plant survival, and the secondary (specialised) metabolism, which fulfils a spectrum of vital roles for plant interactions with the environment [63,121]. Furthermore, an overlap between the primary and secondary metabolism has been identified in plant responses to abiotic stresses [63,121]. In comparison to the highly conserved primary metabolism, secondary metabolic pathways are of a much greater diversity and are triggered at different developmental stages [122,123]. Primary metabolism pathways have been suggested to play some essential roles in salinity stress alleviation, including providing energy required for stress-responsive metabolic mechanisms, modulation of signalling waves, and synthesis of several precursors of the secondary metabolism (Table 1). Secondary metabolism is more dedicated to maintaining a balance between the plant and its environment, and, as such, it responds to salinity stress via a cascade of antioxidants syntheses pathways for ROS scavenging and reduction of oxidative damage (Table 1).

Primary metabolites derived from the tricarboxylic acid (TCA) cycle (organic acids—malic acid, succinic acid, citric acid), glycolysis (sugars), and the shikimate pathways (phenylalanine) serve as precursors for the synthesis of thousands of secondary metabolites (phenolic acids, flavonoids, alkaloids, polyamines) [63,111,134]. These chemically and structurally diverse metabolites represent a vast reservoir of chemistries and biological functions in organismal and cellular metabolic processes involved in abiotic stress responses. Several metabolic studies on different plants have indicated altered primary and secondary metabolite profiles in response to salinity stress (Table 2). Sugars, amino acids, and organic acids and their derivatives are the most altered primary metabolites in osmotic-stressed plants [34,98,139]. Additionally, the perturbed metabolism of secondary metabolites (phenolics acids, flavonoids, phytohormones) has also been detected in several metabolic studies in response to salinity and drought stresses (Table 2) [15,48,140].

Several studies have shown that plants attempt to maintain cellular homeostasis through the production of a broad spectrum of endogenous metabolites that can help mitigate salinity stress. Upon perception of stress, plants activate the signalling network (Section 3) and a multifaceted response, which includes the synthesis of a range of compounds with roles in the alleviation of the negative impacts of salinity. These compounds comprise both primary and secondary metabolites, which have been found to contribute significantly to the survival and maintenance of crop productivity and growth even in saline soils (Table 2). Metabolic analyses of some of the staple food crops, including *Solanum lycopersium* (tomato) [141], *Zea mays* (maize) [144], *Oryza sativa* (rice) [149], and *Brassica* crops (cabbage) [154], under salinity stress have identified alterations in the metabolite levels of the stressed plants, identifying mainly amino acids, organic acids, sugars, polyamines, fatty acids, phytohormones, and phenolic compounds. While most of the amino acids are upregulated in response to salinity, significant increases in the amino acid pool are attributed to proline, phenylalanine, lysine, alanine, and glycine betaine [10,20,134]. In addition, organic acid and fatty acid synthesis were also upregulated in several studies, suggesting their involvement in plant responses to salinity [87,140].

Generally, the accumulation of osmolytes is one of the major salinity tolerance mechanisms that enables plants to maintain low intracellular osmotic potential/stress. The most common osmolytes accumulated under saline conditions include proline, hydroxyproline, glycine betaine, polyamines, sugars, and sugar alcohols [116,155]. These osmolytes improve the plant’s water uptake efficiency, thereby maintaining cell turgor, and reducing ion toxicity and water loss as a result of their osmotic adjustment capabilities [156,157]. Among these, proline and glycine betaine are regarded as the most efficient osmolytes [158]. In addition to its role in osmotic adjustment, proline acts as an antioxidant that scavenges excessive stress-induced ROS and stabilizes proteins, enzymes, membrane structures, and the electron transport system-complex II [116,156,159]. Glycine betaine also stabilizes enzymes and membranes under stress conditions. Additionally, glycine betaine is involved in other metabolic processes, such as the synthesis of alkaloids and stabilization of the peripheral polypeptide of the PS II, thereby maintaining the physiological function of the chloroplast under high salinity [116,159]. Phytohormones have also been reported to affect cell proliferation in root systems and induce root hairs and lateral roots to enhance water and nutrient uptake under saline conditions [156].

Furthermore, phenolic compounds, such as phenolic acids and flavonoids, which are the hub of the secondary metabolism, are found in increased amounts in stressed plants [134]. The biosynthesis of a cascade of phenolics is attributed to their important roles as antioxidants responsible for the scavenging of stress-induced overproduction of free radicals, thereby counteracting the stress resulting from oxidative damage [154,160]. Moreover, phenolics have other secondary roles in stress effect alleviation, and these include the regulation of phytohormones as well as repair of stress-related damage of the photosynthetic equipment [161,162].

Metabolomics studies have further allowed for identification of critical biological pathways with significant roles in conferring salinity tolerance in different plants [69,125]. These pathways include the biosynthesis pathways for the abovementioned stress-responsive metabolites and include the TCA cycle, amino acid-, fatty acid-, polyamine biosynthesis pathways, as well as secondary metabolites biosynthesis, such as the phenylpropanoid and flavonoid biosynthesis pathways [114,163]. Modulations of these metabolic pathways facilitate plants to survive and grow optimally even under high-salinity conditions [164]. Ultimately, metabolomics research provides insight into the rewiring of plant metabolism, providing a link between the genotype and phenotype of plants under salinity stress.

At present, metabolomics studies have proven that there are modulations to several pathways responsible for syntheses of primary and secondary metabolites with significant roles in plant responses in the case of salt-induced metabolic dysfunction [165,166]. However, a significant gap exists in metabolomics research concerning the spatial resolution of the in vivo state of metabolites due to the dynamic nature of the metabolic networks involved [165,167]. This systems biology approach was used in the exploration of salinity tolerance adaptive mechanisms in the legume *Medicago truncatula*, where transcriptional analysis identified the expression of many genes related to stress signalling, and metabolite profiling was employed to explore the different genotype-related responses to stress at the molecular level [168,169,170,171,172,173,174,175]. This global approach contributes to gaining significant insights into the complexity of stress-adaptive mechanisms, as well as aid in the identification of potential targets for crop improvement. In a similar manner, the incorporation of metabolic flux analysis to *omics* data elucidates a metabolic regulation that cannot be detected by metabolic profiling, thus allowing for a broader characterization of the metabolic activities of plants under salinity stress [176,177]. Therefore, a boost in the *omics-*system biology approach is important for achieving a deeper understanding of the systemic mechanisms to develop salinity tolerance for crop plants [178]. Thus, the identification of particular metabolite patterns can be associated with stress tolerance and could point to mechanisms involved in the conference of salinity stress, thereby bridging the gaps in our understanding of the biochemistry behind salinity tolerance.

## 4. Conclusions and Prospective

Plants live under ever-changing conditions that are immensely stressful for plant growth and development and include biotic and abiotic stresses. Salinization is one of the major environmental issues limiting plant productivity, hence threatening food security worldwide. The effects of this stress are aggravated by global warming, climate perturbations, and the exponential populace growth. It is therefore critical to unravel the traits involved in conferring tolerance to such environmental conditions. This review has thus pointed to soil salinization as a detrimental environmental issue and highlighted the problems and impacts of salinization on crop productivity, and further delved into metabolomics as an essential technique to investigate the biochemical processes involved in plant responses to salinity. To conquer and survive against stresses, readjustment of plant metabolic processes is key. At a molecular level, this includes mechanisms, such as signal transduction pathways involving kinases and calcium-mediated mechanisms, stress-responsive gene reprogramming, and biosynthesis of compounds involved in survival define the basic plant defence. In addition to the second messengers (calcium ions and ROS) leading signal transduction pathways, phytohormones, including ABA, SA, JA, and ET, are also synthesised in the early stages of salinity stress. Profiling of the metabolome delves into the biochemical framework that describes the plant-environment interactions, revealing metabolic mechanisms involved in stress alleviation. These include primary metabolism pathways, such as the TCA cycle, several amino biosynthesis pathways, glycolysis, and secondary/specialized metabolism pathways, including the shikimate, phenylpropanoid, and flavonoid synthesis pathways. With a bit of an overlap, metabolic studies have suggested that alterations in the primary and secondary metabolism coherently lead to mild salinity stress alleviation. However, plant metabolomics research is still lacking, more so in plant responses to abiotic stresses. This indicates a prospective research gap that plant scientists should delve into.

## Figures and Tables

**Table 1:** The primary and secondary metabolism pathways involved in plant responses to salinity stress.

Pathways	Description	References
Glycolysis	Multistep reversible metabolic pathway for the production of pyruvate, a substrate for TCA cycle, from glucose	[124,125]
Pentose phosphate pathway	Branches from glucose-6-phosphate and ribose-5-phosphate, it provides substrates for oxidative defence	[126,127]
Tricarboxylic acid cycle	Central pathway for multiple enzyme-catalysed synthesis of organic acids from oxidation of pyruvate Provides energy to sustain anabolic and catabolic reactions involved in stress responses	[125,128]
Amino acid biosynthesis pathways	Several branched pathways in which amino acids are synthesized from their precursors,	[129,130]
Linoleic and other fatty acids pathways	Synthesis of lipids with roles in stress signalling cascades and precursors for phytohormones synthesis	[131,132]
Shikimic pathway	Synthesis of shikimic acid (precursor for phenylalanine synthesis) from the downstream products of the pentose phosphate pathway, erythrose-4-phosphate and phosphoenol	[98,133]
Phenylpropanoid pathway	The primary pathway responsible for production of all phenolics from phenylalanine	[69,134]
Flavonoids biosynthetic pathway	An offramp from the phenylpropanoid pathway, produces a range of flavonoids from 4-coumaroyl-CoA	[135,136]
Polyamine biosynthesis pathways	Synthesis of polyamines (spermine, spermidine, and putrescene) from ornithine, methionine, and arginine	[137,138]

**Table 2:** An overview of metabolism alterations in response to osmotic stresses, and their corresponding roles in plant stress responses.

Metabolite Group	Stress-Responsive Roles	Plant Species	References
	PRIMARY METABOLITES		
Amino acids	ROS scavenging (proline), protein stabilisation and synthesis, redox control	Tomato, Maize and beans	[141,142]
Polyols	Protection of photosynthesis systems, ROS scavenging, protein stabilisation	*Olea europaea*, maize, *Melissa officinalis*	[143,144,145]
Organic acids	Energy production, signalling molecules, antioxidant activities	Rice, soybean	[146,147]
Sugars	Signalling molecules, carbon energy reserve, maintenance of redox homeostasis, osmoprotectants	*Beta vulgaris*, *Oryza sativa*	[148,149,150]
	SECONDARY METABOLITES		
Polyamines	Activation of antioxidant enzymes, regulation of ion channels activity, protein and membrane stabilisation	Legumes, rice	[138,151]
Flavonoids	Radical scavenging, inhibition of pro-oxidant enzymes, stimulate ROS response genes	*Aegilops cylindrica*, *Amaranthus tricolor*	[152,153]
Phenolic acids	Hormonal regulation, antioxidant activity, photosynthetic activity, improve nutrient uptake	*Brassica* crops, *Salvia mirzayanii*, *Thymus* species	[11,136,154]
